# Blood concentration of cyclosporine during early post-transplant period may have influence on the occurrence of chronic graft versus host disease in patients who received allogeneic hematopoietic stem cell transplantation

**DOI:** 10.18632/oncotarget.10988

**Published:** 2016-08-01

**Authors:** Silvia Park, Kihyun Kim, Jun Ho Jang, Seok Jin Kim, Won Seog Kim, Chul Won Jung

**Affiliations:** ^1^ Division of Hematology-Oncology, Department of Medicine, Sungkyunkwan University of School of Medicine, Seoul, Korea

**Keywords:** cyclosporine, blood level, chronic graft versus host disease

## Abstract

**Introduction:**

It has rarely been studied that how the blood level of CsA affect the incidence of chronic GVHD after allogeneic hematopoietic stem cell transplantation (allo-HSCT).

**Methods:**

A total of 183 patients who underwent allo-HSCT from an HLA-matched or haplo matched family donors between 2006 and 2014 were reviewed.

**Results:**

The average monthly CsA blood concentration (CsA^avr^, ng/ml) was calculated in each patient: 0-1, 1-2, and 2-3 months after allo-HSCT. CsA^avr^ at the first month showed significant association with the occurrence of moderate to severe cGVHD in multivariate analysis adjusted for gender, age, total body irradiation, anti-thymocyte globulin, acute GVHD ≥ grade 2 and CsA^avr^ levels of other periods. The risk of cGVHD development was lowest in patients with CsA^avr^ of 200-250 ng/ml when compared to those with CsA^avr^ of ≥ 250 or < 200 ng/ml (p=0.003).

**Conclusions:**

CsA level between 200 and 250 mg/ml during the first month after transplantation was significantly associated with the decreased risk of moderate to severe cGVHD.

## INTRODUCTION

Allogeneic hematopoietic stem cell transplantation (HSCT) is an important curative treatment option for patients with many malignant and non-malignant hematologic diseases [1, 2]. However, substantial risks remain for morbidity and mortality, which are largely attributable to disease relapse [3], graft-versus-host disease [4-6] and infection [7]. Allo-reactivity caused by immunologic reactions between two individuals has great therapeutic benefit through graft-versus-leukemia (GVL) reactions, but is also responsible for development of graft-versus-host disease (GVHD) that can be potentially fatal [8]. Because of treatment related mortality, GVHD, both in its acute and chronic forms, continues to be a major concern following allo-HSCT despite its association with a lower relapse rate [9, 10].

Current approaches for the prevention and treatment of GVHD involve direct blockade of T-cell function [11]. Cyclosporine (CsA), a calcineurin inhibitor (CNI), is one of the most commonly used pharmacologic agents for the prevention of GVHD [12]. CsA is thought to bind to its specific immunophilin, cyclophilin, of immunocompetent lymphocytes, especially T-lymphocytes [13]. This CNI-immunophilin complex inhibits calcineurin, which leads to a reduced function of effector T cells through inhibiting lymphokine production and interleukin release [14]. Despite its widespread use in clinical practice, the dose, target blood level of CsA, and schedule of administration vary among protocols [15]. In addition, although close surveillance, it is not always easy to keep the CsA levels within the desired range. Based on these findings, there have been interesting researches conferring impact of cyclosporine levels on the development of acute GVHD after allo-HSCT, in which higher CsA levels during early post-transplant period contributed to lower risk of acute GVHD [12, 16, 17]. However, the effect of blood level of CsA on the occurrence of chronic GVHD has rarely been studied.

The immune mechanisms eliciting chronic GVHD is differ from those of acute GVHD [18]. Acute GVHD is considered to be the consequent tissue damage initiated from the interaction between donor T cells and host antigen presenting cells, which is amplified by cytokine from damaged tissues and the lipopolysaccharide that leaks through the injured intestinal mucosa [19, 20]. The pathophysiology of chronic GVHD is much more complex and still remains to be determined. Among the possible explanations on how chronic GVHD develops, regulatory T cells (Treg) numbers have been reported to be diminished in chronic GVHD from a series of studies [21-23]. Of note, Tregs are known to be influenced by immunosuppressive therapy [24]. In particular, previous studies demonstrated that calcineurin inhibitors seem to reduce Treg function and T cell proliferation *in vitro* [25-29]. Owing to the fact that CsA has an impact on the number and activity of Tregs [30] and the possible role of T regs on suppression of chronic GVHD, we reasoned that there might be a relationship between blood concentration of CsA and the occurrence of chronic GVHD.

## RESULTS

### Patients’ characteristics

The analysis included 183 patients; their baseline characteristics are listed in Table 1. The median age was 45 years (range, 18-68), and more men than women were included. Acute leukemia was the most common disease and comprised 67.8% (n=124) of all cases. Myelodysplastic syndrome (MDS) and chronic myelomonocytic leukemia (CMML) came next (13.7%, n=25), and severe aplastic anemia, myeloproliferative neoplasm and lymphoma etc. accounted for the rest. Almost all patients received myeloablative conditioning (n=176, 96.2%). Total-body irradiation (TBI)-based conditioning was performed in 52 patients (28.4%), and 53 patients (29.0%) received anti-thymocyte globulin (ATG) during allo-HSCT. Peripheral blood was used as the source of stem cells in all patients. Acute GVHD was occurred in 51 patients (27.9%), 21 of whom experienced acute GVHD of grade 2 or more.

**Table 1:** Baseline characteristics

Characteristics (total n=183)		Number (%)
Age	Median (range)	45 years (18–68)
Sex	Male	102 (55.7%)
	Female	81 (44.3%)
Disease	Acute leukemia	124 (67.8%)
	MDS^*^	25 (13.7 %)
	MPN^**^	13 (7.1%)
	Severe AA	11 (6.0%)
	Others^***^	10 (5.5%)
Conditioning	Myeloablative	176 (96.2%)
	TBI (+)	52 (28.4%)
	ATG (+)	53 (29.0%)
Donor	Matched sibling	179 (97.8%)
	Haplo-identical family	4 (2.2%)
Graft source	Peripheral blood	183 (100.0%)
	Bone marrow	0 (0.0%)
Acute GVHD	Any grade	51 (27.9%)
	Grade 2 or more	21 (11.5%)

Abbreviations: MDS = myelodysplastic syndrome; MPN = myeloproliferative neoplasm; AA = aplastic anemia; TBI = total-body irradiation; ATG = anti-thymocyte globulin; GVHD

* Three chronic myelomonocytic leukemia (CMML) patients were included.

** Six primary myelofibrosis (PMF), one post-essential thrombocythemia myelofibrosis (post ET-MF), and six chronic myeloid leukemia (CML) patients were included.

*** Five non-hodgkin lymphoma (NHL), two paroxysmal nocturnal hemoglobinuria (PNH), one thalassemia, one chronic active EBV infection, and one blastic plasmacytoid dendritic cell neoplasm (BPDCN) patients were included.

### Blood levels of cyclosporine according to post-transplant period

**Figure 1:**
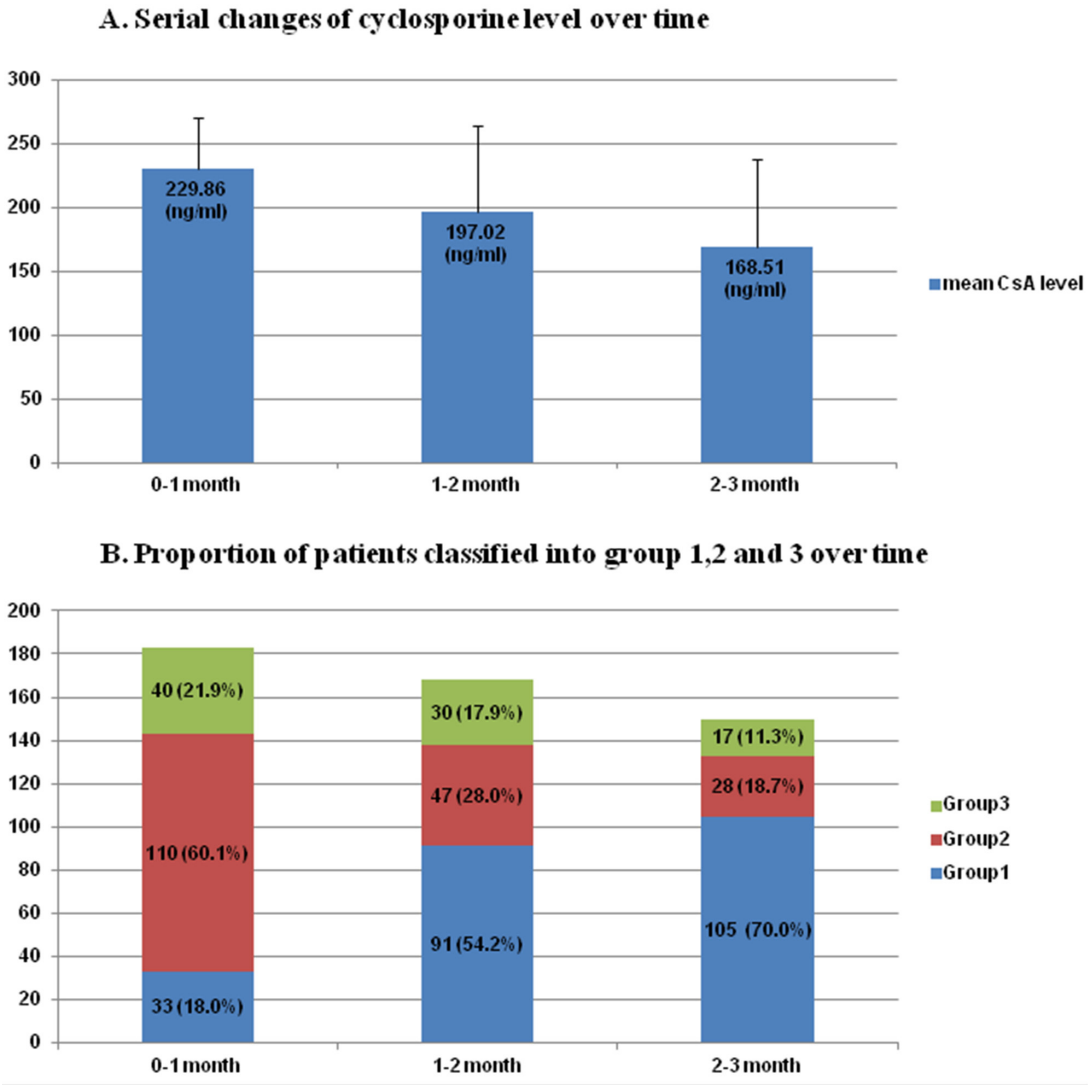
**A.** Serial changes of cyclosporine level over time **B.** Proportion of patients classified into group 1,2 and 3 over time

The mean values of CsA^avr^ were 229.9, 197.0, and 168.5 ng/ml during the first 0-1 month, 1-2 month, 2-3 month after allo-HSCT, respectively (Table 2, Figure 1A). When grouping patients by drug levels, majority of patients were assigned to group 2 (CsA^avr^, ≥ 200 ∼ <250 ng/ml) during the 1^st^ period (n=110, 60.1%). As it went to the 2^nd^ and 3^rd^ period, the average drug levels tended to be lower and the proportion of patients with drug levels corresponding to group 1 is increased: 54.2% during the 2^nd^ period, 70.0% during the 3^rd^ period (Table 2, Figure 1B).

**Table 2:** Blood levels of cyclosporine according to post-transplant period

Post-transplant period	Blood CsA level	Patients, number
0-1 month (available n=183)	Mean, ng/ml (SE)	229.9 (39.9)
	Group 1	33 (18.0%)
	Group 2	110 (60.1%)
	Group 3	40 (21.9%)
1-2 month (available n=168)	Mean, ng/ml (SE)	197.0 (67.7)
	Group 1	91 (54.2%)
	Group 2	47 (28.0%)
	Group 3	30 (17.9%)
2-3 month (available n=150)	Mean, ng/ml (SE)	168.5 (69.3)
	Group 1	105 (70.0%)
	Group 2	28 (18.7%)
	Group 3	17 (11.3%)

Abbreviations: SE, standard error

### The occurrence of moderate to severe chronic GVHD according to CsA blood concentration

**Figure 2:**
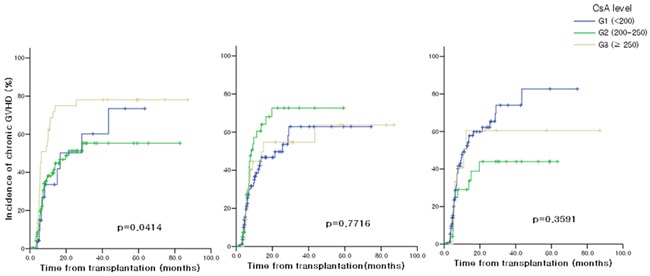
Moderate to severe chronic GVHD according to blood CsA levels **A.** 0-1 month after allo-HSCT **B.** 1-2 months after allo-HSCT **C.** 2-3 months after allo-HSCT

Figure 2 shows the cumulative incidence of moderate to severe chronic GVHD (cGVHD^mod-sev^) according to the blood CsA levels during post-transplant period. Among the drug levels of different periods, the CsA^avr^ level during the first month after transplantation showed significant association with the occurrence of cGVHD^mod-sev^, and the incidence was lowest in group 2 patients. In univariate analysis using Cox proportional hazards regression model, ATG usage was also associated with less occurrence of cGVHD^mod-sev^ (p=0.007) (Table 3). In multivariate analysis adjusted for gender, age, total body irradiation, anti-thymocyte globulin, acute GVHD ≥ grade 2 and CsA^avr^ levels of other periods, the CsA^avr^ level during the first month were proven to significantly be associated with the occurrence of cGVHD^mod-sev^ (p=0.003).

**Table 3:** Univariate and multivariate analyses of cGHVD, DFS, and OS

	cGVHD			DFS			OS		
	univariate	multivariate		univariate	multivariate		univariate	multivariate	
	*P*-value	*P*-value	HR (95% CI)	*P*-value	*P*-value	HR (95% CI)	*P*-value	*P*-value	HR (95% CI)
Sex	0.141	0.274	0.753 (0.453-1.252)	0.634	0.360	0.719 (0.355-1.457)	0.363	0.614	0.856 (0.469-1.563)
Age^*^	0.088	0.859	1.048 (0.621-1.796)	0.877	0.763	1.112 (0.558-2.214)	**0.007**	**0.036**	1.935 (1.045-3.585)
TBI	0.694	0.073	0.594 (0.337-1.050)	0.337	0.452	1.342 (0.624-2.886)	0.663	0.663	0.859 (0.434-1.701)
ATG	**0.007**	0.073	0.462 (0.199-1.076)	**0.032**	0.476	0.683 (0.239-1.949)	0.626	0.531	1.292 (0.579-2.882)
acute GVHD ≥ grade 2	0.901	0.731	1.178 (0.463-2.998)	0.489	0.130	2.308 (0.781-6.818)	0.090	**0.004**	3.647 (1.504-8.843)
CsA^avr^ during 0-1 month^**^	0.051	**0.003**		0.264	1.000		1.000	1.000	
2 vs 1	1	1	0.752 (0.235-2.403)	0.396	1.000	2.128 (0.329-13.78)	1.000	1.000	0.858 (0.242-3.042)
3 vs 1	0.711	0.423	2.294 (0.710-7.414)	1.000	1.000	1.371 (0.172-10.94)	1.000	1.000	0.875 (0.207-3.698)
2 vs 3	0.054	**<0.001**	0.328 (0.144-0.746)	1.000	1.000	1.553 (0.445-5.422)	1.000	1.000	0.981 (0.335-2.874)
CsA^avr^ during 1-2 month^**^	0.795	0.156		0.282	0.264		**0.018**	**0.030**	
2 vs 1	1.000	0.153	2.114 (0.876-5.101)	0.27	0.252	2.566 (0.771-8.538)	0.234	0.09	2.544 (0.925-6.998)
3 vs 1	1.000	1.000	1.198 (0.440-3.258)	1.000	1.000	1.757 (0.445-6.933)	1.000	1.000	0.742 (0.169-3.248)
2 vs 3	1.000	1.000	1.765 (0.626-4.979)	1.000	1.000	1.460 (0.382-5.589)	**0.036**	0.162	3.430 (0.794-14.82)
CsA^avr^ during 2-3 month^**^	0.393	0.105		1.000	1.000		1.000	1.000	
2 vs 1	0.405	0.090	0.387 (0.138-1.087)	1.000	1.000	0.718 (0.181-2.845)	1.000	1.000	0.790 (0.251-2.484)
3 vs 1	1.000	1.000	0.922 (0.298-2.855)	1.000	1.000	1.233 (0.289-5.259)	1.000	1.000	0.995 (0.245-4.046)
2 vs 3	1.000	0.756	0.419 (0.102-1.718)	1.000	1.000	0.582 (0.093-3.657)	1.000	1.000	0.794 (0.145-4.340)

Abbreviations: TBI = total-body irradiation; ATG = anti-thymocyte globulin; OS = overall survival; DFS = disease-free survival; HR = hazard ratio; CI = confidence interval

* Age: <45 and ≥ 45 years of patients were compared.

** Blood drug levels (CsA^avr^) corresponding to Group 1 (CsA^avr^, <200 ng/ml) vs 2 (CsA^avr^, ≥ 200 ∼ <250 ng/ml) vs 3 (CsA^avr^, ≥ 250 ng/ml) were compared.

### DFS and OS according to CsA blood concentration

**Figure 3:**
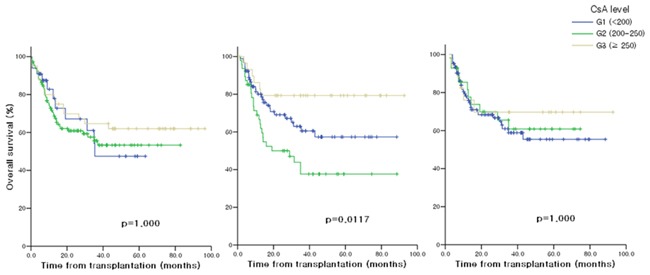
Overall survival according to CsA levels **A.** 0-1 month after allo-HSCT **B.** 1-2 months after allo-HSCT **C.** 2-3 months after allo-HSCT

In the univariate and multivariate analysis for DFS, none of the factors including sex, age, TBI, ATG, acute GVHD ≥ Gr.2 and the CsA^avr^ levels of each period, influenced DFS (Table 3) (Supplementary Figure 1). The univariate analysis for OS revealed that age < 45 years and the CsA^avr^ level ≥ 250 ng/ml during 1-2 month after HSCT showed a significant association with better OS. The result of multivariate analysis for OS was similar to that of univariate analysis showing a significance of age (p=0.036, HR=1.935) and the CsA^avr^ level during 1-2 month (p=0.030), but acute GVHD ≥ Gr.2 was also determined as an independent prognostic factors for worse OS (p=0.004, HR=3.647). Figure 3 demonstrates OS according to the blood CsA levels during post-transplant period.

## DISCUSSION

This study began with clinical question of whether or not there would be an association between post transplant CsA concentration and chronic GVHD. There has already been substantial investigation on how CsA levels affects acute GVHD occurrence, however, relatively little attention has been paid to this question. In this study, we found that average drug levels of first month after transplantation was particularly relevant to the occurrence of chronic GVHD. Of note, chronic GVHD occurred less in group 2 patients, whose average drug levels corresponded to the range of 200-250ng/ml, compared to group 1 or 3 patients. This means it can be disadvantageous for preventing chronic GVHD if the CsA levels are less (group 1) or greater (group 3) than some degree of range. In the analyses of previous studies on acute GVHD, it was observed that higher drug levels within 3 or 4 weeks after HSCT were more effective in prevention of acute GVHD [12, 16, 17], and this is consistent with common concept that prevention of acute GVHD can be achievable by pharmacologic suppression of effector T cell.

Striking finding from the current study is that drug level of 250ng/ml or more were more disadvantageous for preventing chronic GVHD than that of 200-250ng/ml. The result of this study implies that the degree of immune suppression during early post-transplantation period might also influence late allogeneic reactivity such as chronic GVHD. We don’t know exactly how this happened. One of the possible explanations is that the effects of CsA on T cells may vary among the different subsets of T cell population [29]. Actually, T cells consist of several subsets whose responses differ based on antigenic stimuli, activation thresholds, and effector functions [31]. Cyclosporine is the agent that inhibits IL-2 transcription, which leads to fail in T cell proliferation. The role of IL-2 as a growth factor for effector T cells has been well known. However, ironically, IL-2 functions as an indispensable factor for inducing activation-induced cell death (AICD) of activated effector T cells [31, 32], which means IL-2 is necessarily needed to reduce the mass of alloreactive cytopathic effector T cells. In addition, of note, presence of IL-2 is the prerequisite for survival and growth of regulator T cells [33-35]. And, there was a report showing that IL-2 exerts an anti-apoptotic effect on CD4+CD25+ T reg population [36]. Taken together, inhibition of T cell proliferation via IL-2 blockade can be achieved by calcineurin inhibitor such as CsA, and this would be advantageous for prevention of acute GVHD during early post-transplant period. But excessive IL-2 blockade potentially leave relatively large burden of alloreactive T cells through insufficient AICD, and may also hamper survival and growth of regulatory T cells as well as decrease the number of these cells. We cannot assure whether or not suppression of T regs and effector T cell mass over the limit during the early post-transplant period can really instigate a development of chronic GVHD, however, it might be worth considering an impact of excessive IL-2 suppression during early post-transplant period on the late immunologic reaction in stem cell transplanted patients. Other than chronic GVHD, the results in this study indicated that overall survival was influenced by CsA^avr^ level during 1-2 month. Specifically, OS was longest in patients with the CsA^avr^ level ≥ 250ng/ml (group 3), and worst in Group 2 patients (CsA^avr^, ≥ 200 ∼ <250 ng/ml). We cannot assure the precise reason for this finding; but, there might be a causal relationship between the CsA level and the occurrence of acute GVHD. If one experienced acute GVHD, the CsA level would probably be targeted higher. On the contrary, one can had acute GVHD because CsA level was not sufficiently achieved. Given the acute GVHD ≥ Gr.2. was worse prognostic factor for OS, the inseparable connection between CsA level and acute GVHD can affect the results of the analysis.

We acknowledge that there are substantial limitations to the current study. First, institutional strategy for the ATG use has been changed since April 2013. Before then, ATG was not routinely used in matched sibling donor (MSD) transplantation, but only used in transplantation from unrelated donor. Whereas, ATG has actively incorporated into HSCT process even in transplantation from MSD since 2013 based on the recent observations that ATG use was associated with less occurrence of chronic GVHD, but not linked to increasing risk of relapse [37-39]. Although ATG showed significant association for preventing chronic GVHD only in an univariate analysis in this study, it may still be an important factor that can affect chronic GVHD development. In addition, the dose and duration of steroid treatment was not considered in an analysis. In some patients, for example, steroid could have been used for the treatment of acute GVHD, for hormonal replacement in case of adrenal insufficiency, or could have been introduced to patients who were not able to tolerate the use of calcineurin inhibitor. Consequently, heterogeneous distribution of treatment procedures, especially with regard to whether or not patients were treated with ATG and/or steroid that can affect T cell activity as well, might possibly confuse the interpretation of the results. And importantly, the study is limited in its retrospective nature and the patients’ composition of heterogeneous diseases. Lastly, our hypothesis that T regs and effector T cell mass during an early post-transplant period can influence development of chronic GVHD is limited by lack of concomitant experimental data involving T cell subsets, which has to be validated in a prospective manner.

In summary, we found that blood level of CsA during the first month after transplantation was relevant to the occurrence of chronic GVHD. The average drug level between 200 and 250 mg/ml (versus <200 or ≥ 250ng/ml) was significantly associated with the less occurrence of moderate to severe cGVHD.

## MATERIALS AND METHODS

### Patients

One hundred and eighty three patients who received allogeneic HSCT in Samsung Medical Center (Seoul, South Korea) between Sep 2006 and June 2014 were included. Patients who had received methotrexate and cyclosporine for GVHD prophylaxis were identified and retrospectively reviewed. According to institutional strategy, cyclosporine had been used for the prevention of GVHD in matched sibling donor transplantation or in transplantation from haploidentical family donor whereas tacrolimus was the agent used in transplantation from unrelated donor. Consequently, four patients who underwent allo-HSCT from family donors were only included in this study. Patients and donors were HLA typed using high-resolution molecular techniques. Genomic DNA isolated from peripheral blood samples were subjected to sequence-based typing at exons 2, 3 and 4 of HLA-A,B and C genes and at exon 2 of HLA-DRB1 with a commercial SBT kit (AlleleSEQR HLA sequencing kit, Atria Genetics, San Francisco, CA), nucleotide sequencing was performed using an ABI 3730 DNA Sequencer (Applied Biosystems, Foster City, CA) and sequences were analyzed with Assign version 3.5+ software (Conexio Genomics, Applecross, Australia).

### Transplantation procedure and administration of cyclosporine

The conditioning regimen was mainly a combination of fludarabine (30mg/m2/day, 6 days) and busulfan (3.2mg/kg/day, 2 or 4 days) although cyclophosphamide (60mg/kg/day, 2 days) and total body irradiation (TBI) (999cGy, total) combination was primarily used in patients with acute lymphoblastic leukemia. Cyclosporine and methotrexate was used for GVHD prevention, and methotrexate was administered intravenously at 15mg/m^2^ on day 1 and 10mg/m^2^ on day 3, 6, and 11. Cyclosporine was started at 5mg/kg/day as a continuous infusion on day -1, and the dose was reduced to 3mg/kg/day on day 0. Since then, the whole blood cyclosporine concentration was measured every day, and the dose was titrated with a target blood trough level between 150 and 250 ng/ml. The route of CsA administration was switched to oral at a ratio of 1:3 when patients showed acceptable oral intake. After discharge from the hospital at least 3 weeks after transplantation, patients visited outpatient clinic at least once a week for 4 weeks, and at least every 2 week for next 8 weeks with monitoring of CsA blood level at every visit. Blood samples were performed 12 hours after the prior dose, just before the morning dose.

### Blood concentration of cyclosporine and statistical considerations

The blood concentration of CsA was measured by high-performance liquid chromatography-tandem mass spectrometry (HPLC-MS/MS), and the sensitivity of the assay was 5 ng/ml. In the analyses of previous studies on acute GVHD, CsA levels during early post-transplant period, especially within 3 or 4 weeks after HSCT, was a major interest and drug levels were considered usually on a weekly basis [12, 16, 17]. However, because our primary interest was the occurrence of chronic GVHD instead of acute GVHD, we examined drug levels for a longer period of time (for 3 months after HSCT), and the time periods required for the analysis were categorized on a monthly basis. The average CsA blood concentration (CsA^avr^, ng/ml) during a post-transplant period, between 0-1 month, 1-2 months, and 2-3 months after allo-HSCT, was calculated in each patient. The median value of CsA^avr^ was examined in each period, and its correlation with the occurrence of chronic GVHD, overall survival (OS) and disease free survival (DFS) were assessed. Patients were grouped as group 1, 2 and 3 according to average CsA level. When the CsA^avr^ was below 200 ng/ml, they were classified as group 1; group 2 when the level was between 200 and 250 ng/ml, and group 3 if the level was 250 ng/ml or more. Chronic GVHD was determined and scored as mild, moderate or severe according to the National Institutes of Health (NIH) criteria [40]. Chronic GVHD of moderate to severe global severity, which needs systemic immune suppressive therapy, were only accounted for the analysis. The cumulative incidence of moderate to severe chronic GVHD, disease free survival (DFS) and overall survival (OS) according to CsA blood levels were estimated by the Kaplan-Meier method and compared by the log-rank test. The association of moderate to severe chronic GVHD, DFS and OS with clinical characteristics as well as blood CsA level was evaluated using the Cox proportional hazards regression model. P-value and 95% confidence interval (CI) were corrected by Bonferroni's method due to multiple testing. A two-sided P value of <0.05 was considered significant. Statistical analyses were performed using the Statistical Package for the Social Sciences (SPSS) v. 17.0 (SPSS Inc., Chicago, IL, USA).

## SUPPLEMENTARY FIGURE LEGENDS


