# Supplementary material for: Blood concentration of cyclosporine during early post-transplant period may have influence on the occurrence of chronic graft versus host disease in patients who received allogeneic hematopoietic stem cell transplantation
Source: Oncotarget. 2016 Aug 1;7(37):59892–901. doi: 10.18632/oncotarget.10988 (PMC5312356; doi:10.18632/oncotarget.10988)
Supplement: Supplementary file 1 [file oncotarget-07-59892-s001.pdf]

Blood concentration of cyclosporine during early post-transplant period may have influence on the occurrence of chronic graft versus host disease in patients who received allogeneic hematopoietic stem cell transplantation

SUPPLEMENTARY FIGURE LEGENDS

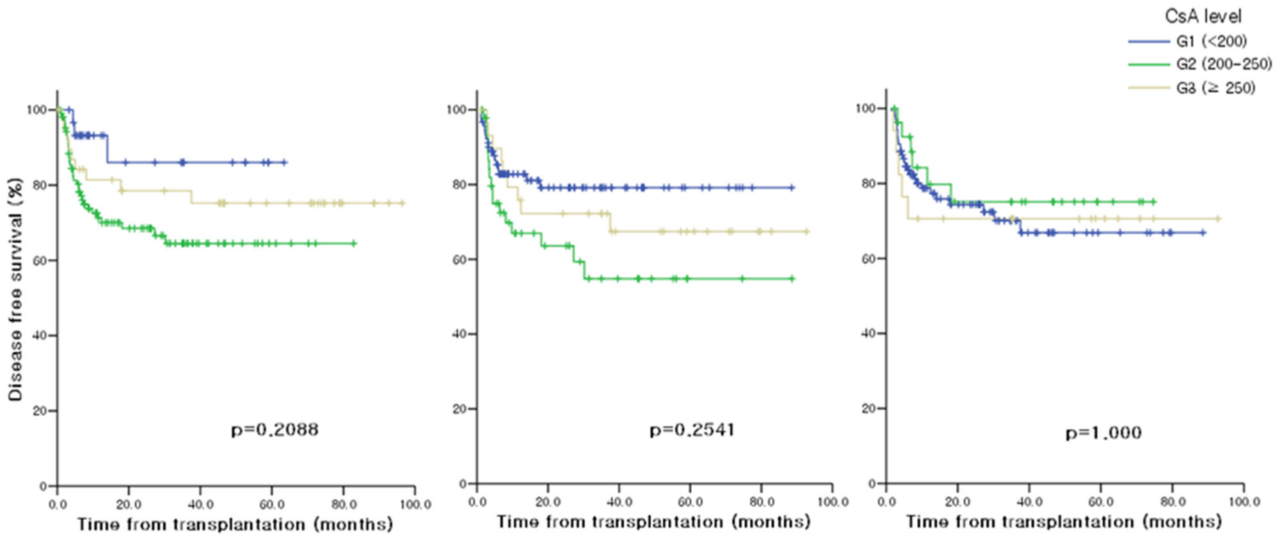

Supplementary Figure 1: Disease free survival according to blood CsA levels
